# Excess mortality in persons with severe mental disorder in Sweden: a cohort study of 12 103 individuals with and without contact with psychiatric services

**DOI:** 10.1186/1745-0179-4-23

**Published:** 2008-10-14

**Authors:** Dag Tidemalm, Margda Waern, Claes-Göran Stefansson, Stig Elofsson, Bo Runeson

**Affiliations:** 1 Department of Clinical Neuroscience, Karolinska Institutet, Division of Psychiatry St. Göran Hospital, Vårdvägen 3, SE-112 81 Stockholm, Sweden; 2 Institute of Neuroscience and Physiology, Section of Psychiatry and Neurochemistry, Sahlgrenska Academy at Göteborg University, Blå Stråket 15, SE-413 45 Göteborg, Sweden; 3 National Board of Health and Welfare, SE-106 30 Stockholm, Sweden; 4 Department of Social Work, Stockholm University, SE-106 91 Stockholm, Sweden

## Abstract

**Background:**

Investigating mortality in those with mental disorder is one way of measuring effects of mental health care reorganisation. This study's aim was to investigate whether the excess mortality in those with severe mental disorder remains high in Sweden after the initiation of the Community Mental Health Care Reform. We analysed excess mortality by gender, type of mental health service and psychiatric diagnosis in a large community-based cohort with long-term mental disorder.

**Methods:**

A survey was conducted in Stockholm County, Sweden in 1997 to identify adults with long-term disabling mental disorder (mental retardation and dementia excluded). The 12 103 cases were linked to the Hospital Discharge Register and the Cause of Death Register. Standardised mortality ratios (SMRs) for 1998–2000 were calculated for all causes of death, in the entire cohort and in subgroups based on treatment setting and diagnosis.

**Results:**

Mortality was increased in both genders, for natural and external causes and in all diagnostic subgroups. Excess mortality was greater among those with a history of psychiatric inpatient care, especially in those with substance use disorder. For the entire cohort, the number of excess deaths due to natural causes was threefold that due to external causes. SMRs in those in contact with psychiatric services where strikingly similar to those in contact with social services.

**Conclusion:**

Mortality remains high in those with long-term mental disorder in Sweden, regardless of treatment setting. Treatment programs for persons with long-term mental disorder should target physical as well as mental health.

## Background

Increased risk of premature death, both from natural and unnatural causes, has been reported for all common mental disorders [1]. Explanations for this include behaviour putting life at risk or aimed at ending life [1], poorer quality of medical care [2-6], side effects of neuroleptics [7-11], heavy smoking [12], substance use [1], unhealthy diet [13], a generally unhealthy lifestyle [14], vulnerability to violence of others [15] and effects of the mental disorder itself [16]. Differences in patterns of mortality between men and women and among different psychiatric diagnostic groups have been observed [1,6,15]. Some causes of premature death in this group are related to medical treatment and the social environment; thus, patterns of death causes may vary over time [1,17].

In many countries, emphasis has shifted from hospital-based to community-based mental health services [18,19]. In Sweden, the latest phase in this process was the Community Mental Health Care Reform which was initiated in 1995 [20]. A major feature of this reform was that the responsibility for supplying supported housing and daily occupational activities to persons with severe mental disorder was transferred from psychiatric to social services. The number of individuals with long-term mental disorder residing in the community has thus increased. Therefore, it has become increasingly important to study community-based, rather than hospital-based, cohorts of persons with mental disorder.

Previous studies of Swedish inpatient samples have reported a more than twofold increase in overall mortality in schizophrenia, affective disorders and other mental disorders, as compared to general population controls [6,21-23].

Obviously, mortality rates and excess mortality may reflect the availability and quality of mental health services. Investigating mortality in those with mental disorder is thus one way of measuring effects of mental health care reorganisation. We wanted to investigate whether the excess mortality in those with severe mental disorder remains high after the initiation of the Community Mental Health Care Reform. The main objective of this reform was to instigate social service interventions, and, as a result, many persons with severe mental illness now receive care from social services rather than psychiatric services. Therefore, we wanted to examine possible differences in mortality between those in psychiatric treatment and those with social service contact. Should we expect lower natural cause mortality in one or the other group? Monitoring of physical health could be better in the psychiatric health services as staff are medically trained. On the other hand, social service staff interact with their clients in daily-life settings where they might offer support regarding access to primary health care and lifestyle changes.

In a previous study we examined predictors of suicide within a large community-based cohort of persons with a long-term and disabling mental disorder [24]. This sample is to our knowledge unique, in that it is a large cohort which includes severely mentally ill both with and without psychiatric contact. In the present study, which is based on the same cohort, we quantified excess mortality due to both natural and external causes compared to the general population. Mortality was examined by gender, type of mental health service and inpatient psychiatric treatment history. Further, mortality was compared in diagnostic subgroups among those with a history of psychiatric inpatient care.

## Methods

### The cohort

The National Board of Health and Welfare, the Stockholm County Council and the Stockholm County Association of Local Authorities conducted a survey in 1997, in order to identify the target population for the Community Mental Health Care Reform. Staff from psychiatry and social services were requested to identify all adult individuals with a long-term disabling mental disorder who were in contact with the services (by personal visit or telephone). At the time of the survey, Stockholm County had 1.8 million inhabitants, of which 1.4 million were aged 18 years or older. This corresponds to about one fifth of the Swedish population 1997. The county contains both rural and urban areas; the latter comprised Stockholm City, the capital and largest city of Sweden (population 730 000 in 1997), and also 24 smaller municipalities (9000 to 84 000 inhabitants 1997).

The inclusion criteria specified residents of Stockholm County aged 18 years or older, with mental disorder causing long-term disability requiring psychiatric care or support from social services. Individuals with mental retardation or dementia and no other mental disorder were excluded. The specified duration of disability was six months or more. These criteria were formulated by the Swedish National Board of Health and Welfare. The survey resulted in a register of 12 267 individuals. Every Swedish citizen has a unique personal identification number. One hundred and sixty-four persons were excluded from the study due to missing data or incorrect identification numbers, yielding a study cohort of 12 103 individuals. Data were collected under the auspices of the government and the study was thus not subject to ethics board review. Social services in six municipalities chose not to participate. Here, data were collected from psychiatric services only. Based on information from other surveys, we estimate that 1000 individuals were missing for this reason. The six municipalities had altogether 225 000 inhabitants aged 18 years or older, corresponding to 16% of Stockholm County's adult population at the time.

### The survey questionnaire

For each individual, staff completed a questionnaire that covered sociodemographic variables and variables related to psychiatric and social service interventions. Questionnaires were completed by both social service staff and psychiatric service staff for 2105 individuals. In these instances, only responses from the social service questionnaire were utilised. This approach was chosen by the National Board of Health and Welfare in the original collection of data, as social service interventions were the main objective of the 1995 Community Mental Health Care Reform.

### The registers

Data were anonymised for linkage and analysis through the use of encryption. Personal identification numbers for the 12 103 cases were encrypted and linked to the Swedish Hospital Discharge Register (HDR) at the Epidemiological Centre of the National Board of Health and Welfare. This register covers all admissions to inpatient care at all hospitals in the country from the mid-1970s. All discharges from psychiatric inpatient care between January 1, 1990, and December 31, 2000 were identified. The diagnostic groups (ICD-10) in this study were derived exclusively from the HDR 1990–2000. Thus, comparisons of SMRs for different diagnostic groups were made only within the subgroup with a history of psychiatric inpatient care (n = 7740). A subject may have received more than one diagnosis. Encrypted personal identification numbers were linked to the Cause of Death Register (the Epidemiological Centre) in order to identify all deaths that occurred in the cohort between January 1, 1997, and December 31, 2000. The Cause of Death Register includes all deaths among persons registered as residents in Sweden at the time of death. Cause of death is determined by a physician. If the cause of death is unclear, an autopsy is performed. Deaths are classified by the ICD-codes for underlying and contributing causes of death. Swedish national registers are generally considered to be of good quality and are regarded as an invaluable resource in epidemiologic and public health research [25].

### Statistics

Standardised mortality ratios (SMRs) and 95% CIs were calculated by a person-years at risk method using PAMCOMP 1.41 [26], for the entire cohort, for the subgroups from psychiatric and social services and for the subgroups with and without psychiatric inpatient care. Person-years at risk 1998–2000 were calculated for each 5-year age group, gender and calendar year. We chose 1998 as the starting point, since cases were entered at different times during 1997. The 95% CIs were calculated assuming a Poisson distribution of the observed number of deaths [27]. The reference population was the population of Stockholm County. The number of excess deaths for each cause of death was calculated by subtracting the expected number of deaths from the observed number of deaths.

## Results

Table 1 describes the cohort at baseline. Few were married or had children under 18 at home. Employment was uncommon (6.6%). A majority (64.5%) resided in own or rented home. Other types of housing included lodging (5.5%), group/supported housing (9.0%), institution (7.4%) and homelessness (2.1%). Information on housing was missing in 11.5%.

**Table 1:** Baseline characteristics in a community-based cohort with severe mental disorder (n = 12 103)

Background variable		Frequency/mean	Per cent^a^
Service provider	Psychiatry	7468	(61.7%)

	Social services only	4635	(38.3%)

Gender	Male	5809	(48.0%)

	Female	6294	(52.0%)

Mean age 1997		46 (sd 14.4)	

Native language	Swedish	9298	(76.8%)

Marital status	Single	9647	(79.7%)

	Married/Cohabiting	1949	(16.1%)

Children under 18		1579	(13.0%)

^a. ^All percentages do not add up to 100, due to missing values.

There were 663 deaths in the entire cohort during the three-year study period, corresponding to 5.7% of the men and 5.2% of the women (Table 2). The number of excess deaths in the total cohort was 417, corresponding to 70% and 56% of all deaths in men and women, respectively.

**Table 2:** Mortality 1998–2000 in a community-based cohort with severe mental disorder, by gender

		Men (n = 5809)				Women (n = 6294)			
**Cause of Death**^a^	**ICD-10 chapter**	**Obs**	**Exp**	**SMR (95% CI)**	**Excess deaths**	**Obs**	**Exp**	**SMR (95% CI)**	**Excess deaths**

**All natural causes**	I-XVIII	255	89.9	2.8 (2.5 – 3.2)	165	278	138.7	2.0 (1.8 – 2.3)	139

Neoplasms	II	31	27.6	1.1 (0.8 – 1.6)	3	54	41.2	1.3 (1.0 – 1.7)	13

Diseases of the circulatory system	IX	113	38.3	3.0 (2.4 – 3.6)	75	133	59.0	2.3 (1.9 – 2.7)	74

Ischaemic heart diseases	IX	66	20.9	3.2 (2.5 – 4.0)	45	56	23.9	2.3 (1.8 – 3.0)	32

Cerebrovascular diseases	IX	18	7.1	2.5 (1.5 – 4.0)	11	33	15.1	2.2 (1.5 – 3.1)	18

Diseases of the respiratory system	X	24	5.7	4.2 (2.7 – 6.3)	18	30	10.1	3.0 (2.0 – 4.3)	20

Diseases of the digestive system	XI	20	4.3	4.7 (2.9 – 7.2)	16	11	5.0	2.2 (1.1 – 4.0)	6

**All external causes**	XX	79	10.3	7.6 (6.0 – 9.5)	69	51	6.8	7.5 (5.6 – 9.9)	44

Accidents	XX	22	4.7	4.7 (3.0 – 7.1)	17	13	3.5	3.8 (2.0 – 6.4)	10

Suicide and undetermined (X60-84, Y10-34)	XX	56	4.9	11.4 (8.6 – 14.8)	51	36	2.9	12.4 (8.7 – 17.1)	33

Suicide (X60-84)	XX	40	3.8	10.6 (7.6 – 14.4)	36	29	2.3	12.5 (8.4 – 17.9)	27

Undetermined intent (Y10-34)	XX	16	1.1	14.3 (8.2 – 23.2)	15	7	0.6	11.9 (4.8 – 24.5)	6

**All Causes**		334	100.2	3.3 (3.0 – 3.7)	234	329	145.5	2.3 (2.0 – 2.5)	183

^a. ^Only causes of death with 15 or more observed deaths in either gender are presented here, to emphasise the more statistically stable results.

Table 2 shows further standardised mortality ratios (SMRs), demonstrating a threefold increase in men and a twofold increase in women for overall mortality, compared to the general population. To emphasise the more statistically stable results, only causes of death with 15 or more observed deaths in either gender are shown in the table. Mortality was significantly increased in almost all these causes of death, in both genders. Additionally, mortality in men was significantly increased in infectious diseases, endocrine diseases, nervous system diseases, urogenital diseases and unspecified diseases (ICD-10 chapter XVIII). Mortality in mental and behavioural disorders was significantly increased for both genders. There were no cause of death categories with decreased mortality. Overall, the number of excess deaths by natural causes (ICD-10 chapter I-XVIII) was almost three times higher than by external causes (ICD-10 chapter XX) (304 cases vs. 113 cases).

The highest SMRs were observed for external causes (ICD-10 chapter XX), especially suicide and undetermined intent, for both genders. The numbers of excess deaths were highest in suicide including undetermined intent (X60-84, Y10-34), closely followed by ischaemic heart disease, for both genders.

The number of excess deaths due to diseases of the circulatory system was particularly high. Common causes of death in this category included ischaemic heart diseases, cerebrovascular diseases, atherosclerosis (I70, ICD-10), pulmonary embolism and heart failure. Pneumonia, emphysema and chronic obstructive pulmonary disease were the most common causes in the respiratory disease category. Gastric and duodenal ulcer, intestinal diseases, alcoholic liver disease and unspecified gastrointestinal haemorrhage dominated in the digestive category.

The causes of death presented in Table 2 were analysed separately for the subgroups reported by social services only and by psychiatry (Table 3). Overall mortality was similar in the two subgroups. SMRs for suicide including undetermined intent were higher for psychiatric services than for social services, but we cannot conclude a significant difference as the confidence intervals overlapped.

**Table 3:** Mortality 1998–2000 in a community-based cohort with severe mental disorder, by service provider

	Psychiatric services (n = 7468)				Social services only (n = 4635)			
	Men (n = 3484)		Women (n = 3984)		Men (n = 2325)		Women (n = 2310)	

**Cause of Death**	**Obs**	**SMR (95% CI)**	**Obs**	**SMR (95% CI)**	**Obs**	**SMR (95% CI)**	**Obs**	**SMR (95% CI)**

**All natural causes**	109	2.5 (2.1 – 3.0)	110	1.8 (1.5 – 2.2)	146	3.1 (2.7 – 3.7)	168	2.1 (1.8 – 2.5)

Neoplasms	11	0.8 (0.4 – 1.4)	28	1.3 (0.8 – 1.8)	20	1.4 (0.9 – 2.2)	26	1.4 (0.9 – 2.0)

Diseases of the circulatory system	49	2.7 (2.0 – 3.6)	50	2.2 (1.7 – 3.0)	64	3.1 (2.4 – 4.0)	83	2.3 (1.8 – 2.8)

Ischaemic heart diseases	30	3.0 (2.0 – 4.3)	26	2.8 (1.8 – 4.1)	36	3.3 (2.3 – 4.5)	30	2.1 (1.4 – 2.9)

Cerebrovascular diseases	7	2.2 (0.9 – 4.4)	9	1.6 (0.7 – 3.0)	11	2.8 (1.4 – 5.0	24	2.5 (1.6 – 3.8)

Diseases of the respiratory system	11	4.4 (2.2 – 7.8)	10	2.5 (1.2 – 4.5)	13	4.1 (2.2 – 7.0)	20	3.3 (2.0 – 5.2)

Diseases of the digestive system	8	3.5 (1.5 – 7.0)	4	1.8 (0.5 – 4.5)	12	6.0 (3.1 – 10.4)	7	2.6 (1.0 – 5.4)

**All external causes**	53	8.8 (6.6 – 11.5)	32	8.9 (6.1 – 12.5)	26	6.0 (3.9 – 8.8)	19	5.9 (3.6 – 9.3)

Accidents	12	4.6 (2.4 – 8.0)	4	2.6 (0.7 – 6.6)	10	4.9 (2.3 – 8.9)	9	4.7 (2.2 – 8.9)

Suicide and undetermined (X60-84, Y10-34)	40	13.6 (9.7 – 18.5)	26	14.1 (9.2 – 20.6)	16	8.2 (4.7 – 13.2)	10	9.4 (4.5 – 17.3)

Suicide (X60-84)	30	13.3 (9.0 – 19.0)	23	15.7 (9.9 – 23.5)	10	6.6 (3.1 – 12.1)	6	7.0 (2.6 – 15.2)

Undetermined intent (Y10-34)	10	14.7 (7.0 – 27.0)	3	7.9 (1.6 – 23.0)	6	13.7 (5.0 – 29.8)	4	19.3 (5.3 – 49.4)

**All Causes**	162	3.3 (2.8 – 3.8)	142	2.2 (1.9 – 2.6)	172	3.4 (2.9 – 3.9)	187	2.3 (2.0 – 2.6)

Causes of death were also analysed for the subgroups with and without psychiatric inpatient treatment during the period 1990–2000 (Table 4). There was greater excess mortality in those with a history of psychiatric inpatient care, both for natural and external causes and in both genders. However, mortality in natural and external causes was significantly increased in both genders compared to the general population also in the group without psychiatric inpatient care. Suicide mortality was substantially lower in those without a history of psychiatric inpatient care.

**Table 4:** Mortality 1998–2000 in a community-based cohort with severe mental disorder, by hospital treatment history

	Psychiatric inpatient care (n = 7740)				No psychiatric inpatient care (n = 4363)			
	Men (n = 3725)		Women (n = 4015)		Men (n = 2084)		Women (n = 2279)	

**Cause of Death**	**Obs**	**SMR (95% CI)**	**Obs**	**SMR (95% CI)**	**Obs**	**SMR (95% CI)**	**Obs**	**SMR (95% CI)**

**All natural causes**	170	3.4 (2.9 – 3.9)	200	2.4 (2.1 – 2.8)	85	2.2 (1.7 – 2.7)	78	1.4 (1.1 – 1.8)

Neoplasms	17	1.1 (0.6 – 1.7)	40	1.6 (1.1 – 2.1)	14	1.2 (0.7 – 2.0)	14	0.9 (0.5 – 1.5)

Diseases of the circulatory system	71	3.3 (2.6 – 4.2)	91	2.6 (2.1 – 3.2)	42	2.5 (1.8 – 3.3)	42	1.7 (1.3 – 2.3)

Ischaemic heart diseases	41	3.5 (2.5 – 4.8)	39	2.8 (2.0 – 3.8)	25	2.7 (1.8 – 4.0)	17	1.8 (1.0 – 2.8)

Cerebrovascular diseases	9	2.3 (1.0 – 4.3)	19	2.1 (1.3 – 3.3)	9	2.8 (1.3 – 5.3)	14	2.3 (1.2 – 3.8)

Diseases of the respiratory system	16	5.2 (2.9 – 8.4)	20	3.3 (2.0 – 5.1)	8	3.1 (1.3 – 6.1)	10	2.5 (1.2 – 4.5)

Diseases of the digestive system	12	4.8 (2.5 – 8.4)	9	3.0 (1.4 – 5.7)	8	4.5 (1.9 – 8.8)	2	1.0 (0.1 – 3.7)

**All external causes**	68	10.6 (8.2 – 13.4)	40	9.6 (6.9 – 13.1)	11	2.8 (1.4 – 5.0)	11	4.2 (2.1 – 7.4)

Accidents	17	6.0 (3.5 – 9.6)	6	2.9 (1.1 – 6.3)	5	2.7 (0.9 – 6.4)	7	5.0 (2.0 – 10.3)

Suicide and undetermined (X60-84, Y10-34)	50	16.1 (11.9 – 21.2)	32	17.4 (11.9 – 24.6)	6	3.3 (1.2 – 7.3)	4	3.7 (1.0 – 9.5)

Suicide (X60-84)	37	15.5 (10.9 – 21.3)	26	17.7 (11.6 – 26.0)	3	2.2 (0.4 – 6.3)	3	3.5 (0.7 – 10.2)

Undetermined intent (Y10-34)	13	18.1 (9.6 – 30.9)	6	16.2 (6.0 – 35.3)	3	7.5 (1.5 – 21.9)	1	4.6 (0.1 – 25.5)

**All Causes**	238	4.2 (3.7 – 4.7)	240	2.7 (2.4 – 3.1)	96	2.2 (1.8 – 2.7)	89	1.5 (1.2 – 1.9)

Observed values were generally higher than expected in all subgroups for all analysed causes of death except neoplasms. For neoplasms, observed and expected values were by and large similar (tables 2, 3, 4).

Mortality was generally more increased in younger ages, regardless of treatment setting. Some of the age stratified results are unstable, due to small numbers (Table 5, 6).

**Table 5:** Mortality 1998–2000, stratified by age, in a community-based cohort with severe mental disorder, by service provider

	Psychiatric services (n = 7468)				Social services only (n = 4635)			
	Men (n = 3484)		Women (n = 3984)		Men (n = 2325)		Women (n = 2310)	

**Age Groups by Cause of Death**	**Obs**	**SMR (95% CI)**	**Obs**	**SMR (95% CI)**	**Obs**	**SMR (95% CI)**	**Obs**	**SMR (95% CI)**

**All natural causes**								

15–39 y	8	4.8 (2.1 – 9.5)	2	1.8 (0.2 – 6.4)	8	8.2 (3.6 – 16.2)	9	15.8 (7.2 – 29.9)

40–89 y	101	2.4 (2.0 – 2.9)	108	1.8 (1.5 – 2.2)	138	3.0 (2.5 – 3.6)	159	2.0 (1.7 – 2.4)

**All external causes**								

15–39 y	24	13.5 (8.7 – 20.1)	6	13.2 (4.9 – 28.8)	8	7.5 (3.3 – 14.9)	4	16.6 (4.5 – 42.6)

40–89 y	29	6.9 (4.6 – 9.9)	26	8.3 (5.4 – 12.1)	18	5.5 (3.3 – 8.7)	15	5.1 (2.8 – 8.3)

**Table 6:** Mortality 1998–2000, stratified by age, in a community-based cohort with severe mental disorder, by hospital treatment history

	Psychiatric inpatient care (n = 7740)				No psychiatric inpatient care (n = 4363)			
	Men (n = 3725)		Women (n = 4015)		Men (n = 2084)		Women (n = 2279)	

**Age Groups by Cause of Death**	**Obs**	**SMR (95% CI)**	**Obs**	**SMR (95% CI)**	**Obs**	**SMR (95% CI)**	**Obs**	**SMR (95% CI)**

**All natural causes**								

15–39 y	15	8.4 (4.7 – 13.9)	8	7.2 (3.1 – 14.2)	1	1.2 (0 – 6.6)	3	5.1 (1.0 – 14.8)

40–89 y	155	3.2 (2.7 – 3.7)	192	2.3 (2.0 – 2.7)	84	2.2 (1.7 – 2.7)	75	1.4 (1.1 – 1.7)

**All external causes**								

15–39 y	31	16.1 (11.0 – 22.9)	9	19.9 (9.1 – 37.7)	1	1.1 (0 – 6.1)	1	4.2 (0.1 – 23.2)

40–89 y	37	8.2 (5.8 – 11.3)	31	8.4 (5.7 – 11.9)	10	3.3 (1.6 – 6.1)	10	4.1 (2.0 – 7.6)

Within the subgroup with a history of psychiatric inpatient care (n = 7740), substance use disorder (F10-19, ICD-10) was the diagnosis with the highest SMRs for natural causes in both men and women (men: 6.1, 95% CI 4.7–7.8; women: 3.9, 95% CI 2.5–5.8). SMRs for external causes were highest for both genders in those with personality disorder (F60-69; men: 20.5, 95% CI 11.9–32.8; women: 29.1, 95% CI 15.5–49.8) and those with substance use disorder (men: 14.7, 95% CI 9.6–21.5; women: 24.6, 95% CI 13.1–42.0). Suicide mortality (including undetermined intent) was especially high in those with a personality disorder (men: 37.6, 95% CI 21.5–61.0; women: 41.7, 95% CI 20.8–74.7) Remaining SMRs for all the analysed mental disorders (including also psychotic disorders (F20-29), mood disorders (F30-39) and anxiety disorders (F40-48)) ranged between 2.0 and 4.3 for natural causes and between 6.3 and 20.0 for external causes. (Data not shown in table.)

## Discussion

To the best of our knowledge, this is the first paper to compare mortality in severely mentally ill with and without psychiatric contact. Mortality in the cohort was at similar high levels as in previous Swedish studies of individuals with severe mental disorder hospitalised during the 1970s, 1980s and beginning of 1990s [6,21-23], regardless of whether treatment was delivered by psychiatric or social services.

Some methodological issues require attention. Firstly, inclusion was determined by service providers and what constitutes a "mental disorder causing long-term disability" may vary in different service settings, which may introduce bias [28]. This approach, however, allowed inclusion of persons without psychiatric contact, which is a major strength of the study. Secondly, diagnostic data are lacking for those with outpatient psychiatric care only. Thirdly, SMRs are strictly speaking only comparable between populations which have exactly the same age distribution. However, research has shown that in practice SMRs may be used to compare different subgroups or cohorts, unless the age distributions are extremely different [29], which is not the case in this study. Fourthly, the results were not adjusted for length of duration of mental disorder. The increased mortality is greatest in the early course of mental disorder, especially in suicide, but possibly also in other causes of death [3,5,22,23,30]. The excess mortality in the cohort is therefore probably somewhat underestimated, due to survivorship bias. This, however, does not affect the conclusions of the study. Fifthly, the quality of the data for potentially confounding socioeconomic variables was insufficient to allow for inclusion in models. It should be noted that a model including socioeconomic variables would have to take into consideration that, e.g., socio-economic status may both lie on the causal pathway from mental disorder to premature death and act as a confounder. Finally, the inclusion criteria were broad, which prevents direct comparison with studies that focus on a specific diagnosis.

SMRs where strikingly similar in those reported by psychiatry or social services, respectively. Longer follow-ups are needed to determine whether differences in mortality in these two groups will emerge over time. Most likely, the increased natural cause mortality in those who had had psychiatric inpatient care indicates an association with severity of the mental disorder. It is also possible that medical comorbidity increases the probability of being admitted for psychiatric inpatient care. Mortality was more pronounced among younger persons, which is consistent with the findings of others [5,22,23,31].

### Causes of death

The number of excess deaths from natural causes was threefold that of external causes; this result is in line with previous studies [1,32-34] and underlines the need for health interventions for this vulnerable group. Reasons behind the increased mortality in ischaemic heart diseases in those with a severe mental disorder include smoking, overeating, physical inactivity and side-effects of neuroleptics, including metabolic syndrome. Another explanation may be that persons with severe mental disorder do not receive adequate care, e.g., revascularisation procedures [4,35-37]. Two recent studies show that the latter cause may be avoidable [38,39]. The pronounced mortality in respiratory and digestive diseases was possibly due to heavy smoking and alcohol use, but could also be related to unhealthy diet, a generally unhealthy lifestyle or ineffective medical care [6,12-14]. Mortality in cancer was not elevated compared to the general population. Several previous studies have reported observed values for neoplasms similar to expected or lower in those with schizophrenia and other mental disorders, despite presence of heavy smoking and other risk factors [1,5,23,40,41]. This has led to speculation about protective biological or behavioural factors, especially in schizophrenia [42]. A recent study found some support for an intrinsic protection in schizophrenia, but the same authors reported an increased risk of colon cancer, especially in those taking antipsychotic medications [43].

### Psychiatric diagnoses and mortality

The high risk of premature death in those with a substance use disorder is well-known [1,44]. Substance use disorders often co-occur with other diagnosed or undiagnosed mental disorders. Treatment of those with concurrent substance use disorder and other mental disorder is considered difficult; validated treatment strategies are lacking [44-48]. Proposed treatment strategies for this group include integrated mental health and substance abuse treatment [44,48,49] or behavioural treatment for substance abuse developed specifically for people with severe and long-term mental disorders [45]. Those with a personality disorder had a particularly high mortality from external causes, compared to the general population. Borderline personality disorder was the strongest diagnostic predictor of suicide within the same cohort [24]. It has been noted that suicide prevention is particularly challenging in individuals with borderline and other personality disorders [50,51].

### Possible interventions

The excess mortality in this group is not only a health care problem, but also a societal problem. It is essential that those with a long-term severe mental disorder actually receive the social support and assistance to which they are legally entitled. Social support can include focus on lifestyle factors and facilitate access to primary care and medical and psychiatric services. At this point it is unclear to what extent an increase in the number of psychiatric beds would affect mortality [52,53].

Causality is often a complex issue, for instance regarding the respective influence of lifestyle factors, lack of appropriate medical care, neuroleptic use and metabolic effects, as well as cardiovascular effects of the disease itself [4,37,54]. It may therefore be difficult to target specific causes of premature death in preventive programs. Consequently, a general, broad range improvement of medical care and social services for this group is possibly the most effective means for reduced mortality on a large scale.

## Conclusion

Excess mortality in those with long-term mental disorder remains high in Sweden, regardless of gender, type of mental health service or diagnosis. Treatment programs for persons with long-term mental disorder should target physical as well as mental health.

## Competing interests

The authors declare that they have no competing interests.

## Authors' contributions

DT co-designed the study, performed the statistical analyses, managed the dataset, and wrote the paper. MW and BR co-designed and cowrote the paper. CGS had the original idea for the study, co-designed it and coordinated management of data. SE was adviser on design and statistics. All authors read and approved the final manuscript.
